# Heat Transfer Analysis between R744 and HFOs inside Plate Heat Exchangers

**DOI:** 10.3390/e24081150

**Published:** 2022-08-18

**Authors:** Anas F. A. Elbarghthi, Mohammad Yousef Hdaib, Václav Dvořák

**Affiliations:** Department of Applied Mechanics, Faculty of Mechanical Engineering, Technical University of Liberec, Stdentská 1402/2, 46117 Liberec, Czech Republic

**Keywords:** R744, plate heat exchanger local analysis, heat transfer analysis, pressure drop analysis

## Abstract

Plate heat exchangers (PHE) are used for a wide range of applications, thus utilizing new and unique heat sources is of crucial importance. R744 has a low critical temperature, which makes its thermophysical properties variation smoother than other supercritical fluids. As a result, it can be used as a reliable hot stream for PHE, particularly at high temperatures. The local design approach was constructed via MATLAB integrated with the NIST database for real gases. Recently produced HFOs (R1234yf, R1234ze(E), R1234ze(Z), and R1233zd(E)) were utilized as cold fluids flowing through three phases: Liquid-phase, two-phase, and gas-phase. A two-step study was performed to examine the following parameters: Heat transfer coefficients, pressure drop, and effectiveness. In the first step, these parameters were analyzed with a variable number of plates to determine a suitable number for the next step. Then, the effects of hot stream pressure and cold stream superheating difference were investigated with variable cold channel mass fluxes. For the first step, the results showed insignificant differences in the investigated parameters for the number of plates higher than 40. Meanwhile, the second step showed that increasing the hot stream pressure from 10 to 12 MPa enhanced the two-phase convection coefficients by 17%, 23%, 75%, and 50% for R1234yf, R1234ze(E), R1234ze(Z), and R1233zd(E), respectively. In contrast, increasing the cold stream superheating temperature difference from 5 K to 20 reduced the two-phase convection coefficients by 14%, 16%, 53%, and 26% for R1234yf, R1234ze(E), R1234ze(Z), and R1233zd(E), respectively. Therefore, the R744 is suitable for PHE as a driving heat source, particularly at higher R744 inlet pressure and low cold stream superheating difference.

## 1. Introduction

A plate heat exchanger (PHE) includes several alternative channels of hot and cold fluid. Each channel is confined between two corrugated plates. PHEs are commonly used in several applications, such as cooling systems, food processing, chemical industries, and power generation due to their heat recovery capability, compactness, and low working fluid charge [1,2,3]. The PHE can be designed as one control volume, called the lumped analysis design or by dividing the control volume into multiple artificial sections, called the local design [4]. The latter approach is preferable, as it considers the phase change within the heat exchanger. The characteristics mentioned above have ensured a wide range of applications for PHEs in the cooling systems, especially for the ejector cooling system. The performance of the ejector cooling systems is susceptible to the working condition. Therefore, it is crucial to analyze the pressure profile in the whole system, particularly in the heat exchangers.

R744 (denoted as CO_2_) has been used widely in the refrigeration system due to its good thermophysical properties and is classified as A1 safety level by ASHRAE [5]. Moreover, the utilization of supercritical R744 overwhelmed the inefficiency problems in refrigeration cycles at high temperatures since it has a low critical temperature [6]. The low critical temperature anticipates smooth variation in the thermophysical properties, resulting in a stable operation of the PHE. Recently, Elbarghathi et al. [5] presented a transcritical R744 refrigeration cycle that utilizes the supercritical phase of R744 at the gas cooler and the subcritical R744 at the evaporator. In these applications, the supercritical R744 temperature is relatively high and distant from the critical point, making it suitable for use as a heat source for different applications of PHE. 

The heat transfer coefficients and pressure drop are crucially essential parameters for the PHE design. In literature, most studies concerned with R744 were performed for channel or tube heat exchangers [7]. Recently, Zendehboudi et al. [6] examined the heat transfer coefficients and pressure drop of supercritical R744 experimentally inside PHE. The authors claimed that the mathematical models proposed in the literature for Nusselt number are unapplicable for their study since these models are intended for heat transfer inside a channel or tube. Moreover, they derived a correlation for the Nusselt number of the supercritical R744 inside the PHE. Their model predicted the Nusselt number accurately, since they considered the effect of buoyancy forces.

On the other hand, the cold stream inside the PHE passes through the liquid, mixture, and gas phases. In the case of the single-phase flow, the forced convection is considered as a heat transfer regime, while for the two-phase flow, the convective boiling regime is used [8]. For the two-phase flow, Longo et al. investigated the vaporization of HFOs inside the PHE by IR thermography [9,10,11,12]. They found that the PHE area covered by the gas-phase increases significantly with the superheat temperature. Moreover, their results showed that the saturation temperature has a negligible effect on the heat transfer coefficients. 

In contrast, more studies were concerned with the single-phase heat transfer coefficients and pressure drop inside the PHE. Amalfi et al. [3,13] presented a review of the literature models for calculating the Nusselt number and the fanning friction coefficient for both single-phase and two-phase flows. Moreover, the authors presented correlations for the Nusselt numbers and the fanning friction coefficients for single-phase and two-phase flows. Their correlations are based on experimental data and include the effect of heat flux and buoyancy forces on the two-phase flow.

The geometrical parameters considerably influence the heat transfer process and pressure drop inside PHE, especially on the flow maldistribution phenomenon. Kumar et al. [2] investigated the effect of geometrical parameters on PHE effectiveness and pressure drop. They found that increasing the chevron angle is a trade-off process as it enhances the effectiveness and flow uniformity, but increases the pressure drop. Moreover, they illustrated that the maldistribution increases with the mass flow rate. On the other hand, Shokouhmand et al. [1] examined the effect of the number of plates on the maldistribution and effectiveness. Their most important remark is that the maldistribution is significant at a high number of plates and low chevron angles.

In this study, the supercritical R744 was proposed as a driving heat source for a plate heat exchanger. A local analysis of the PHE was constructed on MATLAB using the literature correlations integrated with the NIST database for real gases [14]. The following HFOs: R1234yf, R1234ze(E), R1234ze(Z), and R1233zd(E) were considered as the cold fluid flowing upon three different phases. First, the effectiveness, pressure drop, and heat transfer coefficients were investigated at different numbers of plates. Then, the effect of the boundary conditions on the effectiveness, pressure drop, and heat transfer coefficients was examined for the chosen number of plates. The proposed study introduces supercritical CO_2_ as a heat source for recently manufactured eco-friendly refrigerants, which will be helpful for designing and developing refrigeration cycles. 

## 2. System Description

**Figure 1:**
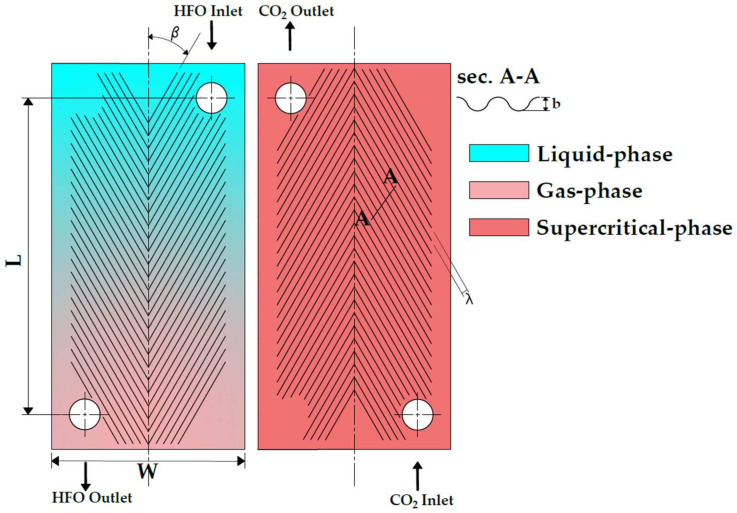
Plate geometry and the flow phases.

Figure 1 illustrates the geometrical parameters of the proposed plate and the fluid phases of both hot and cold streams. The hot stream includes R744, which flows solely in the supercritical phase, while the cold stream includes R1234yf, R1234ze(E), R1234ze(Z), and R1233zd(E), which flow from the liquid-phase to the superheated gas-phase. HFO refrigerants have been chosen due to their low toxicity and non-flammability, and they cope with EU regulations [15]. Moreover, they have a negative slope for the gas saturation line, which leads to a broader range of suitable applications [16]. The proposed plate was chosen with a 60° Chevron angle, in which the maldistribution effect is negligible [2]. The geometrical specifications of the PHE are listed in Table 1.

PHE is considered a one-pass heat exchanger operating at steady-state conditions with negligible heat losses to the surrounding. In addition, each stream is divided into three artificial sections according to the phase change in the cold stream. Since the heat transfer coefficients have weak sensitivity with the saturation temperature, the cold stream had been considered to have an identical temperature profile for all HFOs (inlet temperature of 25 °C and saturation temperature of 87 °C). On the other hand, the hot stream input enthalpy was computed based on the semi-hermetic reciprocating compressor model, manufactured by the Dorin company (Dorin CD1400H) [17]. Moreover, a linear temperature profile is assumed between both streams to estimate the thermophysical properties on the surface between the hot and cold fluids [6]. The thermophysical properties were calculated based on the NIST database for real gases [14]. 

The analytical analysis was carried out by employing the most suitable equations and correlations from the literature into MATLAB by performing the required iterations. First, these correlations were solved solely with a variable number of heat exchanger channels to determine the suitable number of plates based on the estimated effectiveness and total pressure drop. Then, the heat transfer convection coefficients, effectiveness, and pressure drops were investigated at variable hot stream pressures (10, 11, and 12 MPa), different cold stream superheating temperatures (5, 10, 15, and 20 K), and various cold channel mass fluxes. 

## 3. Theoretical Analysis

### 3.1. Data Reduction

As shown in Equation (1), the heat rates of R744 and the cold fluid were assumed to be identical, and this assumption was proved experimentally by Zendehboudi et al. [6].

(1) $$Q_{c}=Q_{h}=Q={\dot{m}}_{c}\left(H_{c,o}-H_{c,\;i}\right),$$

where $H_{c,o}$ and $H_{c,i}$ correspond to the cold stream outlet and inlet enthalpies, respectively.

Since the thermophysical properties change nonlinearly in the supercritical region, the log mean temperature difference (LMTD) cannot be used. Therefore, the actual temperature differences were evaluated as:

(2) $$\Delta T=\frac{Q}{{U\;A}_{\mathrm{eff}}{\;N}_{\mathrm{pl}}},$$

where N_pl_ and A_eff_ are the number of plates and the effective area, respectively. The effective area includes the effect of Chevron corrugation on the total area exposed to the working fluids by multiplying the plate (A_pl_) area by the enlargement factor (∅) [4], as shown below:

(3) $$A_{\mathrm{eff}}=\emptyset \;\mathrm{LW}$$

(4) $$\emptyset =\left(1+\sqrt{1+{\left(\frac{\pi \;b}{\lambda }\right)}^{2}}+\sqrt{\left(1+{\left(\frac{\pi \;b}{\lambda }\right)}^{2}\right)/2}\right)/6$$

The overall heat transfer coefficient (U) was estimated as:

(5) $$U={\left(\frac{1}{h_{c}}+\frac{1}{h_{h}}+\sum f+\frac{t_{\mathrm{pl}}}{\kappa_{\mathrm{pl}}}\right)}^{-1}$$

where f and κ_pl_ are the fouling factor and the plate thermal conductivity, respectively, while h_c_ and h_h_ are the convection coefficient of the cold and hot fluids, respectively. The convection coefficients can be estimated from Nusselt number (Nu) as:

(6) $$\mathrm{Nu}=\frac{{\mathrm{hD}}_{\mathrm{hy}}}{\kappa },$$

where D_hy_ is the channel hydraulic diameter. 

For the liquid-phase and gas-phase flows of the cold stream, the Nusselt number and the fanning friction coefficient were evaluated based on the Martin model [18]. Meanwhile, for the two-phase flow, the correlations proposed by Amalfi et al. [13] were used since their correlations include the effect of the heat flux, buoyancy forces, and turbulence. More importantly, their correlations were based on experimental data for PHEs. Therefore, a group of dimensionless numbers must be computed, which are: Boiling (Bo), Weber (We), Bond (Bd), and Grashof (Gr). The boiling number (Bo) represents the heat flux effect as shown below:

(7) $$\mathrm{Bo}=\frac{q}{G_{\mathrm{ch}}i_{\mathrm{lv}}}=\frac{U\Delta T}{G_{\mathrm{ch}}i_{\mathrm{lv}}}$$

where q, $G_{\mathrm{ch}}$, and $i_{\mathrm{lv}}$ are the heat flux, the channel mass flux, and the heat of vaporization.

The boiling number also indicates the temperature difference between both streams, which is an essential parameter for design purposes. This difference must remain higher than zero since it represents the point when the hot stream temperature becomes close to the vaporization process temperature in the cold stream. The Weber number includes the buoyancy forces effect as:

(8) $$\mathrm{We}=\frac{G_{\mathrm{ch}}^{2}D_{\mathrm{hy}}}{{\sigma \rho }_{\mathrm{avg}}}$$

where σ and $\rho_{\mathrm{avg}}$ are the surface tension and the average density, respectively. The average density was estimated by the two-phase homogenous model as:

(9) $$\rho_{\mathrm{avg}}={\left(\;\frac{X_{m}}{\rho_{v}}+\frac{1-X_{m}}{\rho_{L}}\;\right)}^{-1}$$

where X_m_ is the mean vapor quality of the vaporization process, which is equal to 0.5 when the process starts from the saturated liquid line to the saturated vapor line. 

The Bond number (Bd) represents the turbulence effect as a function of the material thermophysical properties, as shown below:

(10) $$\mathrm{Bd}=\frac{\left(\rho_{l}-\rho_{v}\right){\;\mathrm{gD}}_{\mathrm{hy}}^{2}}{\sigma }$$

On the other hand, the heat transfer coefficients of the hot stream supercritical flow were estimated using the correlation proposed by Zendehboudi et al. [5]. The proposed model includes the effect of buoyancy forces by introducing the Grashof number as shown below:

(11) $$\mathrm{Gr}=\frac{\left(\rho_{w}-\rho_{m}\right)\rho_{m}gD_{hy}^{3}}{\mu_{m}^{2}}$$

where the subscripts w and m represent the property value close to the surface and its mean value, respectively. Therefore, a linear temperature profile was considered between the two streams to estimate properties at the surface, while the mean properties were evaluated at mean temperature as follows:

(12) $$T_{m}=\frac{\sum_{k=0}^{n}T_{h,k}}{n}$$

where k represents each inlet or outlet. This equation evaluates the mean temperature based on the average temperature of all artificial inlets and outlets.

In contrast, the fanning friction coefficient of the hot stream was estimated based on Martin model. For both streams, the total pressure drop was evaluated based on the frictional and the ports pressure drop as follows:

(13) $$\Delta P=0.75N_{\mathrm{ch}}\left(\frac{G_{\mathrm{po}}^{2}}{2{\;\rho }_{i}}-\frac{G_{\mathrm{po}}^{2}}{2{\;\rho }_{o}}\right)+2F\frac{{\mathrm{LG}}_{\mathrm{ch}}^{2}}{D_{\mathrm{hy}}\rho }\;{\left(\frac{\mu_{m}}{\mu_{w}}\right)}^{-0.17},$$

The effectiveness was estimated by the e-NTU method as follows:

(14) $$\mathrm{NTU}=\frac{{U\;A}_{\mathrm{eff}}}{C_{\min}}$$

(15) ε=1−exp(−NTU)

where $C_{\min}$ is the minimum heat capacity of the hot and cold stream as follows:

(16) $$C_{\min}=\min\left({\dot{m}}_{c}{\;\mathrm{Cp}}_{c},{\dot{m}}_{h}{\;\mathrm{Cp}}_{h}\right).$$

### 3.2. Calculation Procedure

**Figure 2:**
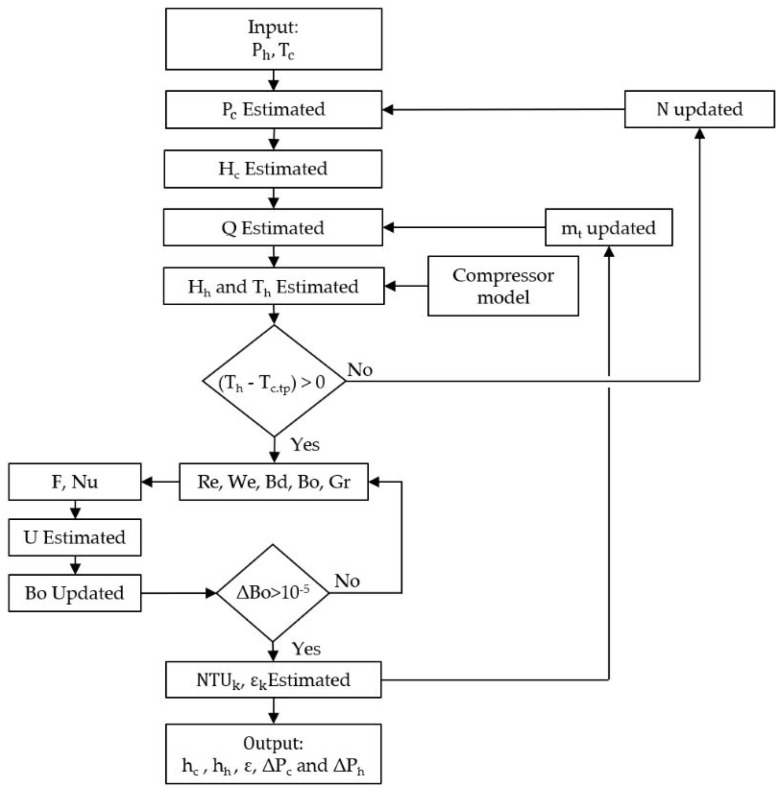
Model flowchart.

The plates were divided into three artificial sections according to the phase change within the cold stream. First, the number of plates effect was investigated at a constant cold stream mass flow rate, superheating temperature difference, and constant hot stream pressure. Then, the suitable number of plates was determined according to its influence on the total pressure drop, effectiveness, and the heat transfer coefficients of both streams. Thereafter, the effect of cold channel mass flux, the cold stream superheating difference, and the hot stream pressure were investigated. The previously mentioned equations were constructed as an iterative MATLAB code integrated with the NIST database. The flow chart in Figure 2 illustrates the calculation steps as follows:

- The inputs are the cold stream inlets, outlets temperature, and hot stream pressure.
- The cold stream pressure was estimated according to the refrigerant name (N) and the specified saturation temperature.
- The cold stream enthalpies were evaluated for the artificial inlet and outlet for each cold stream.
- Then, the heat rate (Q) was estimated for each artificial section by multiplying the total mass flow rate by the enthalpy difference.
- The hot stream temperatures were evaluated using the R744 inlet temperature and the heat rate.
- Thereafter, the dimensionless numbers were evaluated. Re, We, Bd, and Bo were evaluated for the cold stream, while for the hot stream, Re and Gr were evaluated. Since the boiling number requires information from the heat flux and the overall heat transfer coefficient, as shown in Equation (7), a loop was set to iteratively solve Equations (5) and (7).
- Once the boiling number difference condition is achieved, the effectiveness, convection coefficients, and pressure drops will be evaluated and saved. Then, the calculation proceeds with the following flow rate or the number of plates. Finally, the mentioned steps were repeated for different inputs and refrigerants.

## 4. Discussion

### 4.1. Effect of the Number of Plates

**Figure 3:**
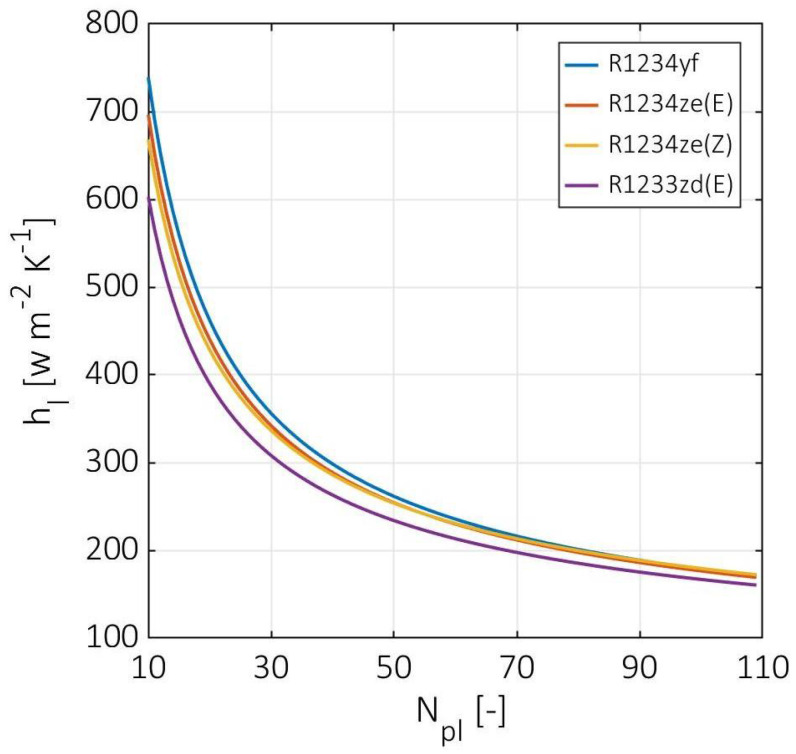
Cold stream liquid-phase convection coefficients at various numbers of plates.

For the number of plates effect, the heat convection coefficients, pressure drops, and effectiveness were investigated at a hot stream inlet pressure of 10 MPa, cold stream superheat temperature difference of 3 K, and cold mass flow rate of 0.1 Kg s^−1^ for four different types of HFOs: R1234yf, R1234ze(E), R1234ze(Z), and R1233zd(E). Figure 3 illustrates the cold stream liquid-phase convection coefficients versus the number of plates from 10 to 109. For all cases, the liquid-phase convection coefficient has the same tendency to decrease from around 700 to 170 W m^−2^ K^−1^. For a number of plates lower than 40, the liquid-phase convection coefficient has a steep slope, and then this slope becomes more horizontal at a higher number of plates. This tendency is due to the change in Reynold’s number as the velocity and the channel mass flux decrease with the increasing number of plates. Moreover, the type of refrigerant has a negligible effect on the liquid-phase convection coefficients, since the viscosities and conductivities differ slightly.

**Figure 4:**
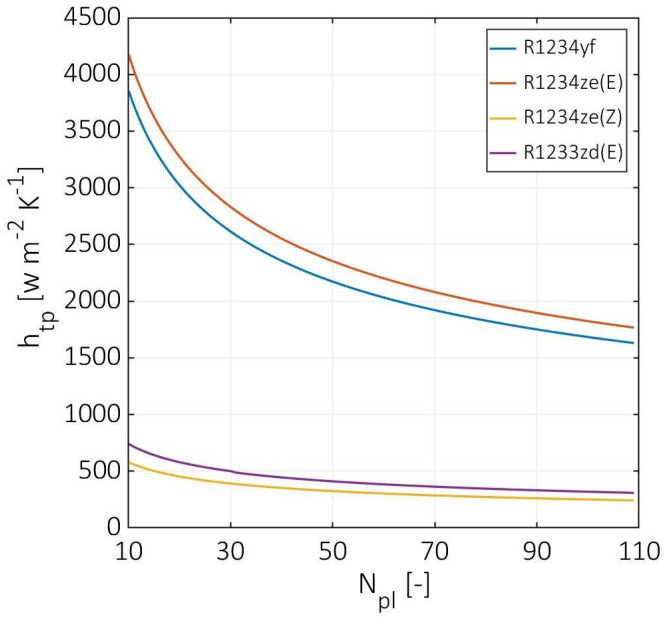
Cold stream two-phase convection coefficients at various numbers of plates.

Figure 4 shows the cold fluid two-phase convection coefficient versus the number of plates. In the case of using R1234yf and R1234ze(E) as the cold fluid, the two-phase convection coefficient decreased from 3856 to 1631 W m^−2^ K^−1^ and from 4175 to 1766 W m^−2^ K^−1^ for R1234yf and R1234ze(E), respectively. In contrast, the two-phase convection coefficient for R1234ze(Z) and R1233zd(E) was approximately five times lower than R1234yf and R1234ze(E). The R1234ze(Z) and R1233zd(E) have considerably lower two-phase convection coefficients due to their higher energy of vaporization, which leads to lower heat fluxes, lower boiling numbers, and lower overall heat transfer coefficients. On the other hand, the two-phase convection coefficient for R1234ze(E) was 8% higher than R1234yf.

In contrast, R1233zd(E) was 28% higher than R1234ze(Z) with values from 740 to 309 W m^−2^ K^−1^ due to the differences in bond number. The bond number is mainly a function of the surface tension, and it is assumed to be constant during the vaporization process for each refrigerant. For all curves, the slope becomes more horizontal with the increasing number of plates due to the decrement in the cold channel mass flux and Reynolds number. However, the estimated two-phase convection coefficients are in good agreement with the experimental data performed by Longo et al. [10,11].

**Figure 5:**
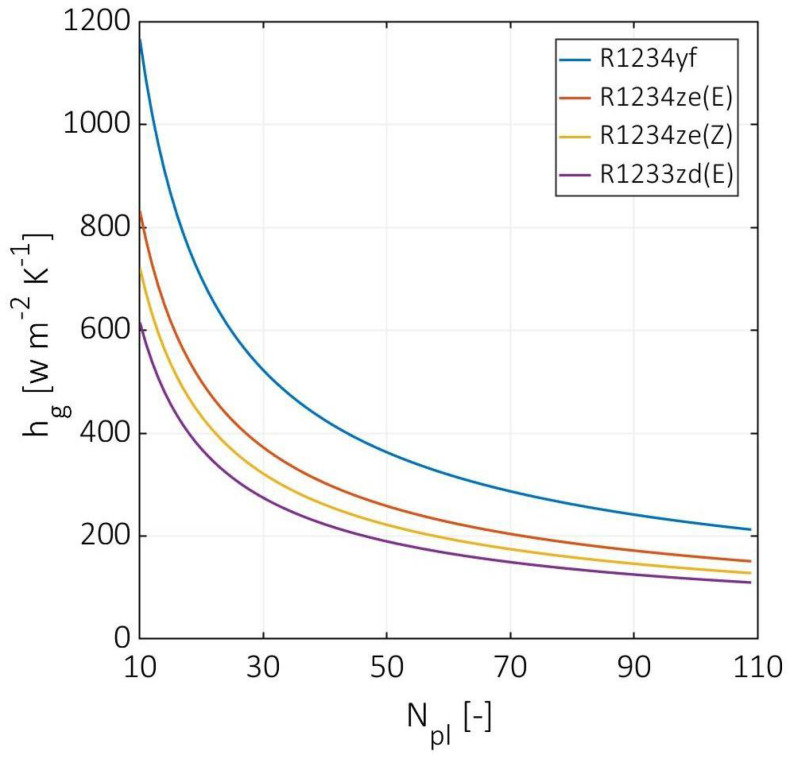
Cold stream gas-phase convection coefficients at various numbers of plates.

Figure 5 demonstrates the cold fluid gas-phase convection coefficient versus the number of plates. The gas-phase convection coefficient for R1234yf was higher than the other used HFOs, decreasing from 1165 to 213 W m^−2^ K^−1^. The other HFOs have a lower two-phase convection coefficient by 28%, 38%, and 47% for R1234ze(E), R1234ze(Z), and R1233zd(E), respectively. Compared to the liquid-phase convection coefficients, higher differences in the gas-phase convection coefficients were observed. This effect is due to considerably lower values of thermal conductivities and viscosities at the gas phase. However, the slopes of the plotted curves for the two-phase convection coefficients become more horizontal as the number of plates increases, especially for the number of plates higher than 60.

**Figure 6:**
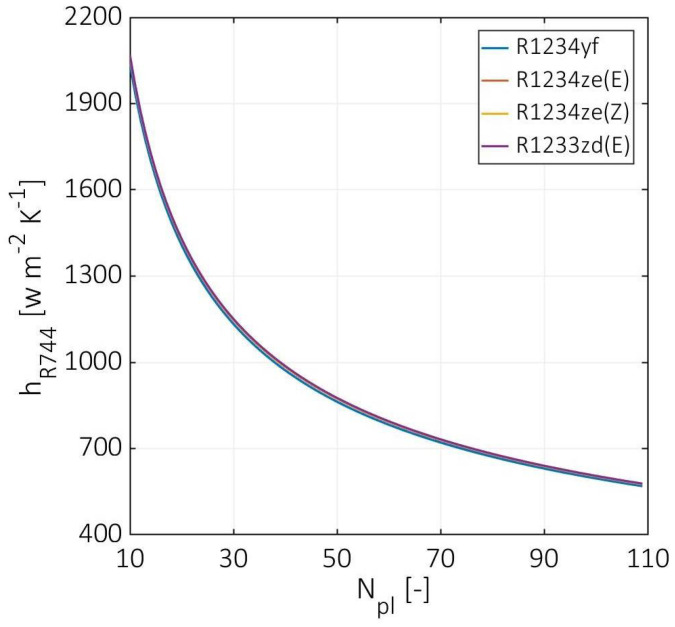
Hot stream convection coefficients at various numbers of plates.

Figure 6 shows the hot stream convection coefficient versus the number of plates. The hot stream convection coefficient has similar curves for all used cold fluids, decreasing from around 2050 to 575 W m^−2^ K^−1^. However, the hot fluid convection coefficient decreases by 62% at the number of plates that is equal to 60, and a slight decline was estimated at a number of plates equal to 110. This decline becomes more stable due to the decrement in Reynolds number and the continuous increase in the Grashof to Reynolds number ratio. The acquired results were calculated at CO_2_ mean temperature of around 90 °C, which is in good agreement with the experiment performed by Zendehboudi et al. [6]. Furthermore, the CO_2_ has a relatively higher temperature than the critical one (31 °C), which provides a more stable PHE operation. 

**Figure 7:**
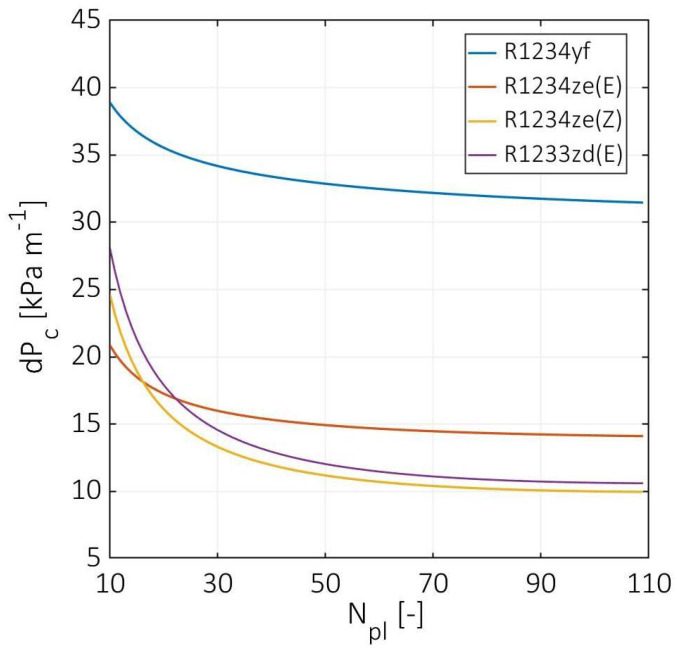
Cold stream total pressure drop at various numbers of plates.

Figure 7 demonstrates the cold stream total pressure drop versus the number of plates. The highest pressure drop was estimated for R1234yf from 38.9 kPa m^−1^ at 10 plates to 31.5 kPa m^−1^ at 109 plates. At a number of plates equal to 10, the total pressure drops to 21, 25, and 28 kPa m^−1^ for R1234ze(E), R1234ze(Z), and R1233zd(E), respectively. In contrast, at a number of plates higher than 60, the R1234ze(E), R1234ze(Z), and R1233ze(E) have a 50%, 65%, and 68% smaller total pressure drop than R1234yf, respectively. On the other hand, R1234ze(Z) and R1233zd(E) have a total pressure drop higher than R1234ze(E) for a number of plates lower than 16 and 22, respectively. First, it should be noted that the two-phase pressure drop dominates the overall pressure drop since it is more turbulent. According to Amalfi et al. [3], the vaporization process is turbulent even at low vapor quality for a bond number higher than 4. Moreover, the two-phase pressure is directly proportional to the Bond number and inversely proportional to the Weber number. Both numbers are strongly dependent on the used refrigerant surface tension. However, the highest pressure was observed for R1234yf since it has the lowest value of Weber number. In contrast, R1233zd(E) had similar values of Weber number, but its pressure drop is considerably lower since it has the lowest bond number, which indicates that it is the least turbulent fluid of the used HFOs. In addition, the R1234ze(E) has the highest Weber and Bond numbers, but its Bond number is considerably higher than the other fluids. Therefore, it has the lowest total pressure drop at a low number of plates and a relatively high-pressure drop at an increased number of plates. The estimated pressure drops are in good agreement with the experiment performed by Longo et al. [10,11] and indicate that an insignificant pressure drop change was observed for a number of plates higher than 40. 

**Figure 8:**
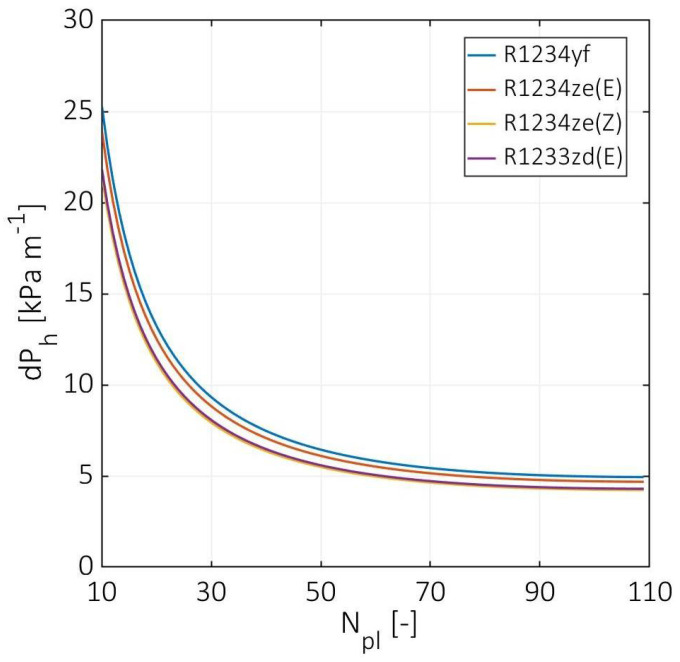
Hot stream total pressure drop at various numbers of plates.

Figure 8 demonstrates the hot stream total pressure drop versus the number of plates. In the case of R1234yf as cold fluid, the hot stream pressure drop decreased from 25 to 5 kPa m^−1^ at a number of plates equal to 10 and 109, respectively. In the case of R1234ze(E), R1234ze(Z), and R1233zd(E), the total hot stream pressure drop declined by 6%, 14%, and 15%, respectively, compared to R1234yf. These differences in pressure drop resulted due to the fact that the CO_2_ temperature drop depends on the cold fluid energy of vaporization. The higher the required vaporization energy, the higher the CO_2_ temperature drop, higher CO_2_ viscosity, and lower Reynolds number. The highest temperature drop was observed for R1234ze(Z), and the lowest was found at R1234yf. Generally, the total hot pressure drop has an insignificant variation at a number of plates higher than 50, in which the hot stream Reynolds number converges, as well. The estimated pressure drop is in good agreement with the data evaluated by Zendehboudi et al. [6].

**Figure 9:**
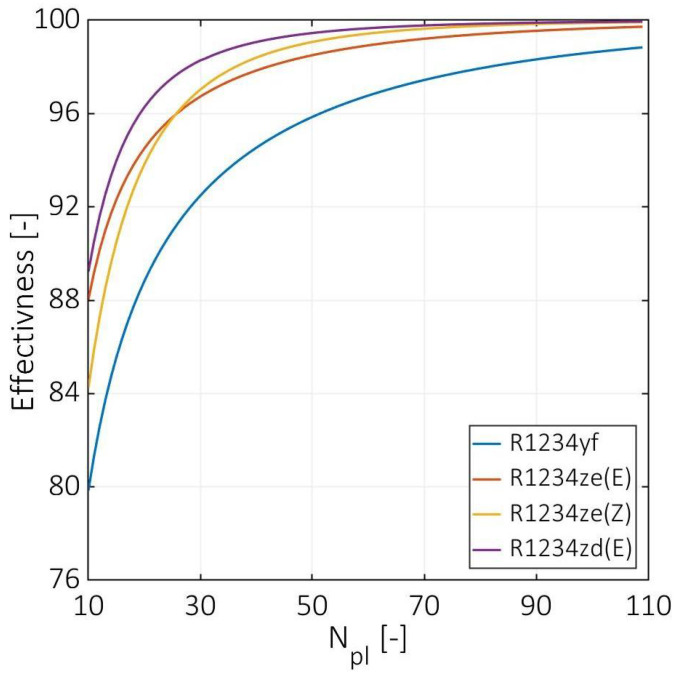
Effectiveness at various numbers of plates.

Figure 9 demonstrates the PHE effectiveness versus the number of plates. R1233zd(E) has the highest effectiveness from 89% to 99%, followed by R1233ze(Z) and R1234ze(E) from 84% and 88% to 99%, respectively, while the lowest effectiveness was estimated for R1234yf from 80% to 99%. These differences in effectiveness are due to the differences in heat capacities between the used cold fluids. Notably, the e-NTU method was used for the effectiveness calculations, and the minimum heat capacity was observed for the cold fluid for all investigated cases. Consequently, R1234yf has the lowest effectiveness since it has the highest heat capacity, and R1233zd(E) has the highest since it has the lowest heat capacity. Although the heat capacity for R1234ze(E) is higher than the heat capacity for R1234ze(Z), an intersection appears between the two curves at a low number of plates. This intersection can be justified since the R1234ze(E) has the highest overall heat transfer coefficient in the two-phase region. However, the estimated data are in good agreement with the study performed by Shokouhmand et al. [1]. The authors reported that for a number of channels higher than 50, an insignificant variation was observed in the effectiveness, which is also justified by the current study for the effectiveness, total pressure drops, and heat transfer coefficients. 

The previously mentioned results prove that CO_2_ can be used as a heating source for different types of recently produced HFOs at a wide range of plates. The following sections illustrate the effect of different boundary conditions: The hot stream pressure effect and the cold stream superheating temperature effect at various cold channel mass fluxes. In contrast, the impact of the cold stream saturation temperature was ignored as it is insignificant, as reported by Longo et al. [10,11]. For the following sections, the plate heat exchanger is considered to have 40 plates, since at a higher number of plates, insignificant variations were observed for effectiveness, pressure drop, and heat transfer coefficients.

### 4.2. Effect of Hot Stream Inlet Pressure

**Figure 10:**
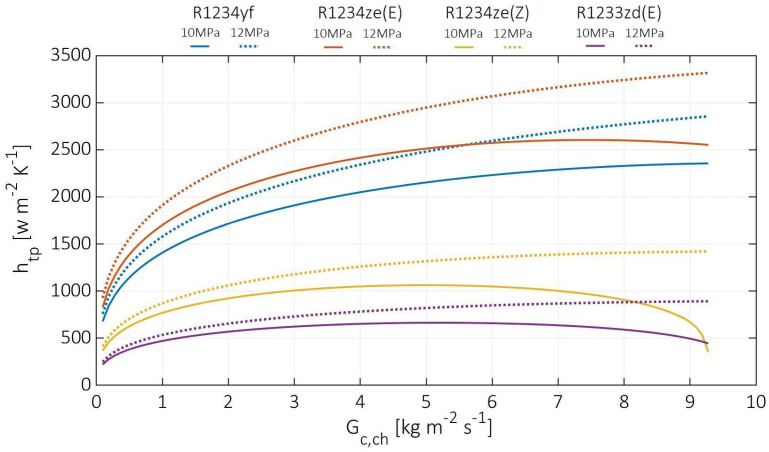
Cold stream liquid-phase and gas-phase convection coefficients at various cold channel mass fluxes.

Figure 10 demonstrates the cold stream two-phase convection coefficients at various cold channel mass fluxes from 0.1 to 9.3 Kg m^−2^ s^−1^, at hot stream inlet pressures of 10 and 12 MPa, and cold stream superheating temperature difference of 3 K. At 12 MPa hot stream pressure, R1234ze(E) has the highest two-phase convection coefficient increasing from 927 to 3315 W m^−2^ K^−1^. In contrast, the other cold fluids have heat transfer coefficients by less than 14%, 57%, and 73% at the highest channel mass flux, and less than 18%, 56%, and 76% at the lowest channel mass flux for R1234yf, R1234ze(Z), and R1233zd(E), respectively. A similar tendency was revealed at 10 MPa hot stream pressure; the R1234ze(E) has a concave curve for the two-phase convection coefficient, increased from 829 to peak at 2601 W m^−2^ K^−1^ then decreased to 2551 W m^−2^ K^−1^. Similar concave curves appeared for R1234ze(E) and R1233zd(E), but with convection coefficients lower by 56%, 73%, and 86%, 83% at the lowest and highest channel mass flux, respectively. Meanwhile, for R1234yf, the two-phase convection coefficient at 10 MPa increased continuously from 927 to 3315 W m^−2^ K^−1^. As previously mentioned, R1234ze(E) has the highest two-phase convection coefficient since it possesses the highest bond number. Generally, the two-phase convection coefficient is higher at higher pressure due to the elevated values of heat flux and boiling number. In addition, the concave shape of the curve is due to the significant decrease in the hot stream temperature and the convergence of the overall heat transfer coefficient to a constant value. At the peak, the overall heat transfer coefficients do not change significantly. Then, the significant reduction in the two-phase convection coefficient occurs when the CO_2_ temperature gets closer to the cold stream saturation temperature, leading to a considerable decrease in the heat flux and boiling number, as shown in Equation (7).

On the other hand, the effect of hot stream pressure on the liquid-phase and gas-phase was insignificant with slight smooth variation, unlike the case for the vaporization process. Moreover, the estimated results justify that the CO_2_ can be used as a heat source with a good range of hot stream pressures. Lower pressures than 10 Mpa for the hot stream were not investigated in this study to maintain the hot stream temperature as high as possible than the cold stream saturation temperature, which provides a wider range of applicable cold flow rates. At the same time, the saturation temperature of the cold stream is considered relatively high to adapt to a wider range of applications, such as ejector cooling systems, where it requires a relatively high temperature inside the ejector mixing chamber to avoid condensation.

### 4.3. Effect of Superheat

**Figure 11:**
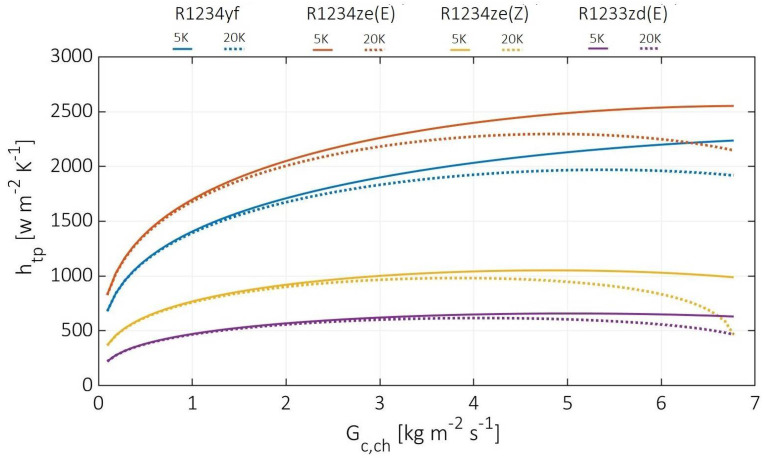
Cold stream two-phase convection coefficient at various cold channel mass fluxes and different superheating temperature differences.

Figure 11 demonstrates the cold stream two-phase convection coefficients at various cold channel mass fluxes from 0.1 to 6.8 Kg m^−2^ s^−1^, at hot stream inlet pressures of 10 MPa, and cold stream superheating temperature difference of 5 and 20 K. At the 5 K cold stream superheating difference, the highest two-phase convection coefficients were observed for R1234ze(E) from 826 to 2553 W m^−2^ k^−1^. At the same time, it was lower for other cold fluids by 18%, 56%, and 73% at 0.1 Kg m^−2^ s^−1^ cold channel mass flux, and lower by 12%, 61%, and 75% at 6.8 Kg m^−2^ s^−1^ cold channel mass flux for R1234yf, R1234ze(Z), and R1233zd(E), respectively. Meanwhile, at 20 K cold stream superheating difference, the two-phase convection coefficient decreased maximally by 16%, 14%, 53%, and 26% for R1234ze(E), R1234yf, R1234ze(Z), and R1233zd(E), respectively. Compared to the hot stream pressure effect, the difference in convection coefficients due to the superheating difference is relatively small. However, these differences occurred due to lower heat fluxes in the two-phase region, since the gas phase covered areas that were significantly increased with the superheating temperature difference [9]. Moreover, the concave curves appeared at 20 K since it corresponds to a higher CO_2_ temperature drop. According to the estimated two-phase convection coefficients, the CO_2_ can operate as a hot fluid for PHE on a wide range of superheating temperatures, making it applicable for high-temperature applications. 

## 5. Conclusions

R744 is commonly used with applications that require supercritical fluid since it has a relatively low critical temperature. Therefore, this study investigates the utilization of R744 as a driving heat source for a plate heat exchanger. The theoretical model was constructed using a MATLAB code integrated with the NIST database of real gases. First, the effect of the number of plates on the effectiveness, pressure drop, and heat transfer coefficients were investigated. Then, the impact of boundary conditions was estimated at 40 plates, in which after 40 plates, insignificant variations were observed in the studied parameters. 

Based on the following conclusions, CO_2_ can be used efficiently as a heat source for plate heat exchangers with different eco-friendly refrigerants.

- The estimated results at various numbers of plates are in good agreement with data from the literature for both cold and hot streams. Moreover, the effectiveness, pressure drops, and heat transfer coefficients vary smoothly or slightly at different hot stream pressures, cold fluid superheating temperatures, and cold channel mass fluxes.
- The cold stream liquid-phase and gas-phase convection coefficients decrease with the increasing number of plates by 40%, when the number of plates changed from 40 and 50 to 109 for liquid-phase and gas-phase, respectively.
- The cold stream two-phase convection coefficients decrease with the increasing number of plates. This decline in the convection coefficient becomes insignificant for the number of plates higher than 40 and lower by 31% when the number of plates changed from 40 to 109. Moreover, the two-phase convection coefficients were more sensitive to the hot stream inlet pressure than the cold stream superheating temperature difference.
- The CO_2_ convection coefficients are almost identical regardless of the used cold fluid. Moreover, slight differences were observed with the changing hot-stream pressure. In addition, when the number of plates changed from 40 to 109, the CO_2_ convection dropped by 34%, which is relatively low compared to the variation at a lower number of plates.
- The two-phase flow dominates the cold stream pressure drop since it is more turbulent. Moreover, the pressure drop in the two-phase region is influenced mainly by the turbulence (Bond number) and the surface tension forces (Weber number), which are a function of the used fluid type since the Reynolds numbers were identical for the used cold fluids. On the other hand, the CO_2_ pressure drop has a similar tendency regardless of the used fluid. However, for both streams, an insignificant variation in the pressure drop was observed at a number of plates higher than 40.

## Figures and Tables

**Table 1:** Geometrical characteristics of PHEs.

Geometrical Parameter	Value
Effective flow length L	485 mm
$\mathrm{Plate}\;\mathrm{thickness}\;t_{\mathrm{pl}}$	0.6 mm
$\mathrm{Port}\;\mathrm{diameter}\;D_{\mathrm{po}}$	55 mm
Corrugation angle β	60°
Plate Pitch Pi	2.8 mm
Mean Channel Gap b	2.2 mm
Plate width W	245 mm
Corrugation pitch λ	6.8 mm

## Data Availability

All data are available from the authors on request.
